# Well-Orientation Strategy Biosynthesis of Cefuroxime-Silver Nanoantibiotic for Reinforced Biodentine™ and Its Dental Application against *Streptococcus mutans*

**DOI:** 10.3390/molecules26226832

**Published:** 2021-11-12

**Authors:** Sanaa M. F. Gad El-Rab, Amal A. Ashour, Sakeenabi Basha, Amal Ahmed Alyamani, Nayef H. Felemban, Enas Tawfik Enan

**Affiliations:** 1 Department of Biotechnology, Faculty of Science, Taif University, Taif 21974, Saudi Arabia; a.yamani@tu.edu.sa; 2 Department of Botany and Microbiology, Faculty of Science, Assiut University, Assiut 71516, Egypt; 3 Department of Oral and Maxillofacial Surgery and Diagnostic Sciences, Oral Pathology Division, Faculty of Dentistry, Taif University, Taif 26571, Saudi Arabia; a.a.ashour@tudent.edu.sa; 4 Department of Preventive and Community Dentistry, Faculty of Dentistry, Taif University, Taif 26571, Saudi Arabia; sakeena@tudent.edu.sa; 5 Preventive Dentistry Department, Faculty of Dentistry, Taif University, Taif 26571, Saudi Arabia; nfelemban@tudent.edu.sa; 6 Dental Biomaterials, Faculty of Dentistry, Taif University, Taif 26571, Saudi Arabia; enasenan275@mans.edu.eg; 7 Dental Biomaterials, Faculty of Dentistry, Mansoura University, Mansoura 35511, Egypt

**Keywords:** antimicrobial activity, Biodentine™, Cefuroxime-ROE-AgNPs, *Rosmarinus officinalis* extract

## Abstract

Dental caries results from the bacterial pathogen *Streptococcus mutans* (*S. mutans*) and is the maximum critical reason for caries formation. Consequently, the present study aims to evaluate the antibacterial activity of a newly synthesized nanoantibiotic–Biodentine formulation. The silver nanoparticles (ROE-AgNPs) were biosynthesized from the usage of *Rosmarinus* *officinalis* L. extract (ROE) and conjugated with cefuroxime to form Cefuroxime-ROE-AgNPs. Using Biodentine™ (BIOD), five groups of dental materials were prepared, in which Group A included conventional BIOD, Group B included BIOD with ROE-AgNPs, Groups C and D included BIOD with Cefuroxime-ROE-AgNPs at concentrations of 0.5% and 1.5% cefuroxime, respectively, and Group E included BIOD with 1.5% cefuroxime. The synthesized ROE-AgNPs or Cefuroxime-ROE-AgNPs were characterized for conjugating efficiency, morphology, particle size, and in vitro release. Minimum inhibitory concentration (MIC) of the cefuroxime, ROE-AgNPs, and Cefuroxime-ROE-AgNPs were additionally evaluated against cefuroxime resistant *S. mutans*, which furthered antibacterial efficacy of the five groups of dental materials. The UV-Visible spectrum showed the ROE-AgNPs or Cefuroxime-ROE-AgNPs peaks and their formation displayed through transmission electron microscopy (TEM), X-ray diffraction (XRD) pattern, and Fourier transforms infrared (FTIR) analysis. The end result of Cefuroxime-ROE-AgNPs showed conjugating efficiency up to 79%. Cefuroxime-ROE-AgNPs displayed the highest antibacterial efficacy against *S. mutans* as compared to cefuroxime or ROE-AgNPs alone. Moreover, the MIC of ROE-AgNPs and Cefuroxime-ROE-AgNPs was detected against *S. mutans* to be 25 and 8.5 μg/mL, respectively. Consequently, Cefuroxime-ROE-AgNPs displayed that a decrease in the MIC reached to more than three-fold less than MIC of ROE-AgNPs on the tested strain. Moreover, Cefuroxime-ROE-AgNPs/BIOD was employed as a novel dental material that showed maximum antimicrobial activity. Groups C and D of novel materials showed inhibitory zones of 19 and 26 mm, respectively, against *S. mutans* and showed high antimicrobial rates of 85.78% and 91.17%, respectively. These data reinforce the utility of conjugating cefuroxime with ROE-AgNPs to retrieve its efficiency against resistant *S. mutant*. Moreover, the nanoantibiotic delivered an advantageous antibacterial effect to BIOD, and this may open the door for future conjugation therapy of dental materials against bacteria that cause dental caries.

## 1. Introduction

*S. mutans* has been demonstrated as a causative bacterium for dental caries, as it ferments carbohydrates to organic acids. These acids cause a lower pH, leading to increased enamel solubility that is manifested in the form of dental caries [1]. Moreover, nanoscale characters can be used for targeted therapeutic delivery and dental applications. Nanoparticle-conjugated biomaterials had been applied in various dentistry and biomedical fields because of their mended biological functionalities and physicochemical and mechanical properties [2]. There are many types of dental material, but BIOD is a must-have product when it comes to vital pulp therapy and the treatment of deep caries. BIOD is among the most recent tricalcium silicate cements that has high biocompatibility and bioactive properties, allowing its application as a permanent substitution of dentin. The material provides a high marginal sealing ability and protection of the pulp tissue through induction of reparative dentin formation [3]. BIOD can be applied without dentin surface preparation, etching, or bonding, as it bonds to tooth structure through micromechanical retention to the dentinal tubules. Previous investigations revealed that the cement can be applied in injured pulp tissue because of its bioactive nature, which enables it to stimulate odontoblasts differentiation [4]. The unique properties of the cement allow its wide use for pulp capping, pulpotomy, and endodontic treatment, with results that are comparable to those of mineral trioxide aggregate (MTA) [5]. Different in vitro and in vivo research confirmed that BIOD is efficient in restorative and pediatric dentistry and in endodontics, as it has strong mechanical properties [6,7,8].

The motive for restorative dental materials that have antimicrobial features has stimulated the introduction of materials containing various antibacterial agents. Amalgam, zirconia, gold alloy, titanium alloy, dental ceramic, and dental resin have been applied as dental restorative materials. These materials are applied for treating dental caries or filling tooth cavities, although the addition of antibiotics to BIOD might also additionally enhance its antibacterial efficacy [9].

**Figure 1:**
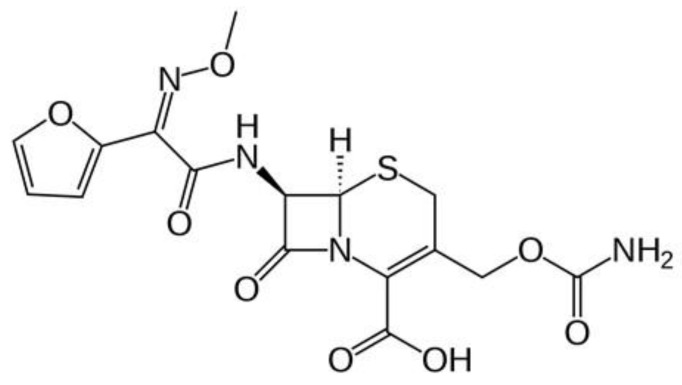
Cefuroxime structure.

Cefuroxime (Figure 1) is similar to cefaclor in that it is consider a second generation cephalosporin. Cephalosporin antibiotics have similar actions to penicillin derivatives, similar to ampicillin, which is used as an alternative to penicillin in patients with allergic reactions and when erythromycin is contraindicated [10]. Cefuroxime appears to be an efficacious bactericidal by affecting the synthesis of the cell wall in bacteria [11] and is used because of its stability with metallic nanoparticles [12,13,14]. Cefuroxime demonstrated that cephalosporin is one of the most effective antibiotics against *S. mutans* isolates [15].

Silver nanoparticles (AgNPs) are exceptionally explored as they are easy to synthesize and are one of the used noble metallic nanoparticles in fields such as catalysis, bio-nano materials, and medicine because of their long-term antibacterial activity and low bacterial resistance in addition to high thermal stability and a high surface area to-volume-ratio [16,17]. Previously, the authors have proven that the usage of plant extracts serve as a low-cost and time-efficient method for biosynthesis of well-characterized AgNPs on a small to large scale [18,19,20]. The cultivated *Rosmarinus officinalis* L. (*R. officinalis* L.) has been extensively applied in traditional medicine. Ghaedi et al. [20] confirmed that the polyphenol and proteins compounds of ROE act as reducing components to form size-controlled ROE-AgNPs. The mechanism of action of AgNPs is unknown, and the suggested mechanisms have been primarily based on their nano-size, which enables the AgNPs to penetrate the microbial cell wall/cell membranes by sulfur-containing proteins or thiol groups, damaging the microbial DNA and leading to cell death [16,17]. Studies have additionally proven that AgNPs conjugate the efficacy of antibiotics against various microbes [21,22]. Researchers have studied the AgNPs applications in several areas regarding antimicrobial efficacy of AgNPs and conjugating effects, especially its application in conjugation with dental biomaterials [23,24,25]. In vitro research has recorded that the addition of metallic nanoparticles into dental materials inhibits oral bacteria growth [23,24,26].

Despite its unique properties as a restorative material, the ability of BIOD to prevent secondary caries is questionable. The present study aims to evaluate the antibacterial activity of a newly synthesized nanoantibiotic-Biodentine formulation against resistant *S. mutans* that cause dental caries.

## 2. Material and Methods

### 2.1. Materials

The current study was performed using one dental cement as shown in Table 1 [27], Biodentine™ (Septodont, Saint Maur des Fosses, France), and two antimicrobial agents, ROE-AgNPs, and cefuroxime (VETRANAL^®^, analytical standard, Sigma-Aldrich, Saint Louis, MO, USA). Accordingly, the two agents were added to the BIOD powder in different combinations (Cefuroxime/BIOD, ROE-AgNPs/BIOD, Cefuroxime-ROE-AgNPs/BIOD) before mixing with the liquid.

### 2.2. Biosynthesis of ROE-AgNPs with Its Conjugation to Cefuroxime and Determination of Conjugation Efficiency

The fresh leaves of *R. officinalis* L. plant from Taif region were collected and washed with sterilized distilled water, and aqueous extract was prepared (10 g/100 mL H_2_O) at 80 °C to obtain reducing and stabilizing agents. Moreover, the ROE was centrifuged and filtered through filter paper (Whatman no. 1, Sigma-Aldrich, Saint Louis, MO, USA). Consequently, the filtrate was used immediately for the synthesis of ROE-AgNPs. Fifty milliliters of 1 mM AgNO_3_ (Sigma-Aldrich, Saint Louis, MO, USA) was mixed with 25 mL of ROE on a magnetic stirrer. The thorough mixing of the reaction mixture was ensured at temperature 80 °C, pH 8 for 2 h, after which it was allowed to settle at room temperature. Accordingly, the change in color to dark brown from using ROE indicated ROE-AgNPs formation [6,7]. ROE-AgNPs were freeze-dried at −60 °C for 24 h at a pressure of 0.011 mbar.

The Cefuroxime-ROE-AgNPs were prepared by mixing cefuroxime with ROE-AgNPs as powders (1:1 mg/mL and 3:1 mg/mL, in a solution of phosphate buffer pH 7.4), followed by incubation overnight, and in the final step, Cefuroxime-ROE-AgNPs were centrifuged at 15,000× *g* for 10 min [28]. Moreover, dialysis of Cefuroxime-ROE-AgNPs against the deionized water was performed using a 10 kDa membrane of cellulose overnight to remove any un-reacted substances. Moreover, the Cefuroxime-ROE-AgNPs were freeze-dried at −60 °C for 24 h at a pressure of 0.011 mbar. The sample was stored to further characterization. Conjugation efficiency of cefuroxime with ROE-AgNPs was calculated spectrophotometrically at 274 nm wavelength [29] according to the following equation: conjugating efficiency of Cefuroxime-ROE-AgNPs (%) = (total amount of cefuroxime − free cefuroxime in the supernatant/total amount of cefuroxime) × 100.

### 2.3. Characterization of ROE-AgNPs and Cefuroxime-ROE-AgNPs

Characterization of ROE-AgNPs and Cefuroxime-ROE-AgNPs was performed using the following methods:

#### 2.3.1. UV-Visible Spectrum

UV–Vis spectrometer (Shimadzu UV-1650 pc spectrophotometer, Tokyo, Japan) was used to record absorbance in the range of 250–700 nm to monitor the rate of cefuroxime, ROE-AgNPs and Cefuroxime-ROE-AgNPs formation.

#### 2.3.2. Transmission Electron Microscopy

The TEM analysis of ROE-AgNPs and Cefuroxime-ROE-AgNPs sample was performed using the TEM model JEOL 100CX II (Tokyo, Japan) with an accelerating voltage of 100 kV (Assiut Electron Microscope Unit, Assiut, Egypt). Consequently, each nano-sample of TEM analysis was prepared by placing a drop of the suspension onto carbon-coated copper grids and allowing it to dry on the grid for 4 min. The shape and size of ROE-AgNPs and Cefuroxime-ROE-AgNPs were determined from a TEM micrograph [30,31].

#### 2.3.3. X-ray Diffraction Analysis

The ROE-AgNPs and Cefuroxime-ROE-AgNPs nature and size were determined using Shimadzu XRD (Shimadzu XD-3A, Tokyo, Japan). The radiation used was Cu Kα (0.154 nm) at 35 nm with a scanning rate of 2°/min and 40 kV. The nanoparticle size was determined using the formula of Debye–Scherrer [32].

#### 2.3.4. Fourier Transforms Infrared Analysis (FT-IR)

FT-IR was determined using a spectrometer (Shimadzu IR-470, Tokyo, Japan) over 4000–500 cm^−1^ [22].

### 2.4. Preparation of Specimens and Grouping

The disc-shaped BIOD specimens were intended for the current research using a split Teflon mold (3 mm in height and 6 mm in diameter) [33,34]. The powder and liquid components of the cement were portioned and mixed according to the manufacturer’s instructions, placed in the molds, allowed to sit for 24 h, and then were removed from the mold and were sterilized using ultraviolet radiation for 30 min. The prepared specimens (*n* = 150) were classified into 5 main groups (30 specimens each) as shown in Table 2:

Then, each of the main groups was tested for antimicrobial efficacy against *S. mutans* at three-time intervals of 1 day, 1 week, and 3 weeks. All samples of five groups were performed in five replicates, and the test was repeated two separate times.

### 2.5. Determination of Drug Release

To measure the cefuroxime release from Cefuroxime-ROE-AgNPs/BIOD, the samples were immersed into 5 mL of phosphate-buffered saline (PBS) at pH 7.4 and incubated at 37 °C for up to 96 days. A UV-Vis spectroscopy (Shimadzu UV-1650 pc spectrophotometer, Tokyo, Japan) was used to determine the concentration of cefuroxime in release medium using a constructed calibration curve. The cefuroxime release (%) was obtained by the equation: *C* (%) = *B*/*A* × 100, where *C* is the cefuroxime release (%), *B* is the total amount of cefuroxime released in the solution, and *A* is the amount of the loaded cefuroxime in Cefuroxime-ROE-AgNPs/BIOD samples.

### 2.6. Bacteria and Growth Conditions

*S. mutans* bacterial strains was taken from our previous study by Enan et al. [35]. This strain was an ESBL-producing bacteria strain that displayed complete resistance to cefuroxime. Before applying the antibacterial test, fresh inoculum of *S. mutans* was prepared in Brain Heart Infusion (BHI) medium (Oxoid Ltd., London, UK), adjusted with the help of BHI to the famous standard (i.e., 1.5 × 10^8^ CFU/mL), and the culture was incubated for 18 h at 37 ± 2 °C.

### 2.7. Determination of Minimum Inhibitory Concentration (MIC) and Minimum Bactericidal Concentration (MBC) of Cefuroxime, ROE-AgNPs, or Cefuroxime-ROE-AgNPs

To determine the MIC and MBC, the broth micro-dilution method recommended by Clinical Laboratory Standards Institute (CLSI) was used [36]. The MIC evaluation was performed on a 96-well plate. Two hundred microliters of cefuroxime (128 mg/mL), ROE-AgNPs (75 mg/mL) or Cefuroxime-ROE-AgNPs (64 mg/mL) was inoculated into the 6 wells in column 1. One hundred microliters of Mueller Hinton broth (MHB) (Oxoid Ltd., London, UK) was placed into the wells in each row. Thereafter, 100 μL of cefuroxime, ROE-AgNPs, or Cefuroxime-ROE-AgNPs solution was taken from column 1 and was serially diluted along the row until column number 10. Thereafter, 5 μL of the bacterial cultures (1.5 × 10^8^) was inoculated into each well containing the MHB medium except column number 10, which was considered blank. Moreover, phenol red dye (2 mg/mL) was prepared, and 50 µL of dye was added to each well to evaluate the viability of bacteria. The plate of the 96-well was incubated and the red color of bacterial cell viability was detected after an incubation time of 24 h at 37 °C. Any color change was observed. No color indicated no bacterial growth, while red color indicated bacterial growth. The minimum concentration of each sample at which no growth or no color was observed was considered as the MIC value. However, after inoculation of 5 μL from each well on the surface of the agar medium plates, MBC was evaluated. The incubation of bacterial culture plates was obtained at 37 °C for 24 h. Finally, the MBC of cefuroxime, ORE-AgNPs, or Cefuroxime-ORE-AgNPs was defined as the lowest concentration of an antimicrobial agent killing the majority (99.99%) of bacterial inoculums. Since the MIC of cefuroxime, ORE-AgNPs, or Cefuroxime-ORE-AgNPs is the ability of inhibitory status, it is possible that if the antimicrobial agent is removed, the bacteria will begin to grow again.

### 2.8. Assessing the Antimicrobial Activity

The antimicrobial efficacy of each BIOD specimen disk of each group against *S. mutans* was displayed according to the Kirby–Bauer method [37] under growth conditions using Mueller Hinton agar (MHA) (Oxoid Ltd., London, UK). Each specimen disk of each group was placed on the MHA medium that was inoculated with *S. mutans*. The clear zone of inhibition around the specimen discs was determined. All samples of each group were performed in triplicate, and the test was repeated five separate times.

### 2.9. Determination of Antibacterial Rate Using Colony-Forming Units (CFU)

Each specimen of the group (A, B, C, D and E) was placed in a 15 mm diameter glass bottle with a flat bottom surface and incubated in 0.5 mL of bacterial *S. mutans* suspension of 1 × 10^6^ cells/mL in 20 mL growth medium (MHB) for 24 h at 37 °C. Each 100 µL of the sample was placed on an MHA plate, separately, and was spread and incubated for 24 h at 37 °C. The antibacterial activity of each specimen was expressed as the CFUs in different incubation times. In addition, the bactericidal ratio (as antibacterial activity) was calculated as the following equation: *AR* = (*CS* − *TS*)/*CS* × 100, where *AR* is an antibacterial rate (%), *CS* is the average colonies number in the control sample (CFU/sample), and *TS* is the average colonies number in the testing samples (CFU/sample). The control sample, 0.5 mL bacterial suspension of 1 × 10^6^ cells/mL in broth medium, was incubated without specimens. Each colonization test was run in triplicate and repeated five separate times.

### 2.10. Formation of Biofilm and Anti-Biofilm Activity of BIOD Material

The anti-biofilm activity of BIOD specimens and biofilm formation of *S. mutans* were analyzed according to the method of Souza et al. [38], with some modifications. In brief, each specimen of the BIOD groups was placed in 24-well plates containing *S. mutans* inoculum, with 2 mL of BHI-B and 5% Sucrose. The 24-well plates were subsequently incubated at 37 ± 2 °C under anaerobic conditions for 3 d to form mature biofilms. After incubation, each BIOD sample was washed gently with distilled water. SEM of each BIOD sample with *S. mutans* biofilm was fixed in fixation solutions of SEM analysis [39]. For SEM, samples of BIOD were placed on stainless-steel holders, sputter-coated with a thin layer of gold, and examined by JOEL SEM (JSM-5400LV, Tokyo, Japan at Electron Microscope Unit, Assiut, Egypt). SEM examinations of BIOD samples were analyzed. Five specimens were examined for each BIOD group.

### 2.11. Statistical Analysis

The difference in mean was assessed using ANOVA, followed by Tukey post hoc analysis. All statistical tests were two-sided, and the significance level was set at *p* < 0.05. Analysis was performed using the Statistical Package for Social Science version 17 (SPSS Inc., Chicago, IL, USA).

## 3. Results

### 3.1. Biosynthesis of ROE-AgNPs with Its Conjugation to Cefuroxime and Determination of Conjugation Efficiency

Herein, ROE-AgNPs have been biosynthesized by reducing AgNO_3_ to ROE-AgNPs using ROE. The formation of ROE-AgNPs in the medium was indicated by a change in color from pale yellow to dark brown after 2 h at pH 8 due to reducing and stabilizing agents in ROE. In addition, cefuroxime was conjugated to ROE-AgNPs to form Cefuroxime-ROE-AgNPs nanoantibiotics. In the present study, the conjugating efficiency of Cefuroxime-ROE-AgNPs was detected to be 79.86%.

### 3.2. Characterization of ROE-AgNPs and Cefuroxime-ROE-AgNPs

#### 3.2.1. UV–Vis Spectroscopy of ROE-AgNPs

**Figure 2:**
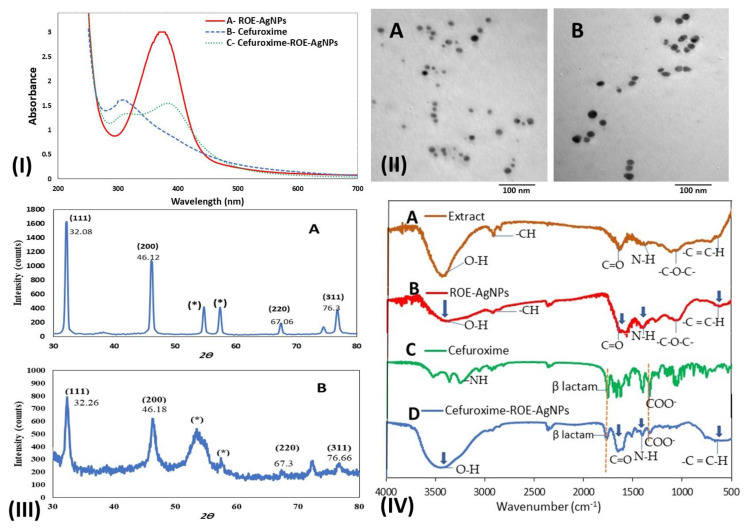
(**I**) Biosynthesis of AgNPs. Notes: UV-Vis spectra of ROE-AgNPs (A) conjugated with cefuroxime (B) to form Cefuroxime-ROE-AgNPs (C). (**II**) TEM images of the ROE-AgNPs synthesized using *R. officinalis* extract (A) and Cefuroxime-ROE-AgNPs (B). (**III**) XRD patterns of the synthesized ROE-AgNPs (A) and Cefuroxime-ROE-AgNPs (B); “*” reveal to residual materials and the numbers in brackets represent the planes of Ag face-centered cubic crystal structures. (**IV**) FTIR spectra of *R. officinalis* extract (A), the synthesized ROE-AgNPs (B), Cefuroxime (C), and Cefuroxime-ROE-AgNPs (D).

As shown in Figure 2I, UV-Vis spectroscopy of ROE-AgNPs showed a peak of 400 nm, and cefuroxime showed a peak of 274 nm, while Cefuroxime-ROE-AgNPs nanoantibiotic showed peaks at 400 and 274 nm for ROEAgNPs and pure cefuroxime, respectively.

#### 3.2.2. Transmission Electron Microscopy

The TEM particle shape and size analysis of ROE-AgNPs and Cefuroxime-ROE-AgNPs showed a formation of spherical particles to oval-shaped nanoparticles (Figure 2(IIA,B)). Moreover, the average size of ROE-AgNPs and Cefuroxime-ROE-AgNPs was estimated to be 8.7 ± 0.57–15.9 ± 1.15 nm and 14.2 ± 0.8–23.3 ± 1.3 nm, respectively.

#### 3.2.3. X-ray Diffraction Analysis

The XRD pattern of ROE-AgNPs (Figure 2(IIIA)) and Cefuroxime-ROE-AgNPs (Figure 2(IIIB)) showed four major peaks at 2θ values of 32.08°, 46.09°, 67.06°, and 76.3°, 32.26°, 46.18°, 67.3°, and 76.66°. These characteristic peaks may be attributed to reflection planes (111), (200), (220), (300), and (311) of face-centered cubic crystalline (FCC) structures of pure Ag^0^ (Figure 1III). The sharpness, strong intensity, and narrow width of ROE-AgNPs diffraction peaks in the XRD pattern (Figure 2(IIIA)) indicated that the synthesized ROE-AgNPs is well crystallized, while broad peaks of Cefuroxime-ROE-AgNPs in the XRD pattern (Figure 2(IIIB)) showed a rise in its size because of the attachment of cefuroxime. According to the Debye–Scherrer analysis, the particle size of ROE-AgNPs and Cefuroxime-ROE-AgNPs was calculated to be 8.2–16 and 13.9–24 nm, respectively.

#### 3.2.4. Fourier Transforms Infrared Analysis

The FTIR was used for characterization of the *R. officinalis* L., ROE-AgNPs, Cefuroxime-ROE-AgNPs, and cefuroxime. Absorbance bands were observed in the region from 4000 to 500 cm^−1^ (Figure 2IV). FTIR spectrum of ROE appeared as major absorption peaks at 3400, 2860, 1620, 1360, 1080, and 597 cm^−1^ that revealed to stretching peaks of O-H, -CH, stretch C=O, N-H amine, -C-O-C- and -C=C-H, respectively (Figure 2(IVA)).

FTIR spectrum of ROE-AgNPs appeared as major absorption peaks at 3330, 2860, 1590, 1360, 1080, and 597 cm^−1^ that revealed stretching peaks of O-H, -CH, C=O, N-H amine, -C-O-C and -C=C-H, respectively (Figure 2(IVB)). In *R. officinalis* L., -OH and -NH of phenolics and proteins were involved in the formation of ROE-AgNPs through reducing Ag^+^ to Ag^0^ (Figure 2(IVB)). The groups at 3330, 2860, 1080 cm^−1^ were associated with the structure of saponin (-OH, -C-H-, and -C-O-C-), respectively, and indicates the presence of residue saponin on the surface of the ROE-AgNPs (Figure 2(IVB)).

Cefuroxime in Figure 2 (IVC) can be interpreted as peaks stretching of (COO^−^) at 1640 and 1390 cm^−1^ respectively, (-NH) bending vibration at 1540 cm^−1^ and the band of (-NH) stretching at 3350 cm^−1^. A peak at 1778 cm^−1^ confirms the presence of a carbonyl group (-C=O) of β lactam stretching in standard cefuroxime.

Upon mixing cefuroxime with ROE-AgNPs (Figure 2(IVD)), the peaks of the carboxylic group in cefuroxime shifted from 1620 to 1681 cm^−1^. This end result suggests that the carboxylic group of cefuroxime binds with the NH^+^ group of ROE-AgNPs to form an amide group of Cefuroxime-ROE-AgNPs.

### 3.3. Determination of Drug Release

**Figure 3:**
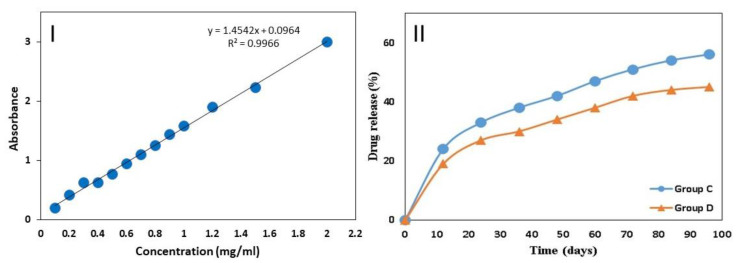
(**I**) External standard linear curve of cefuroxime with limit of detection (LOD = 0.198298). (**II**) In vitro drug release profile Cefuroxime-ROE-AgNPs/BIOD. Group (C) C—99% BIOD with 1% Cefuroxime-ROE-AgNPs nanontibiotic, (1:1) and Group (D) D—98% BIOD with 2% Cefuroxime-ROE-AgNPs nanontibiotic (1:3).

Figure 3 shows that the cefuroxime release (%) from Cefuroxime-ROE-AgNPs/BIOD, group C and D revealed a two-step release: The fast first-step release, which is the release of cefuroxime (%) from Cefuroxime-ROE-AgNPs/BIOD, in which groups C and D reached to 24% and 19% within the first 10 days. The slow second-step release reached almost to 56% and 45%, respectively, after 96 days. The release of cefuroxime (%) was calculated using an external standard linear curve of cefuroxime.

### 3.4. MIC and MBC of Cefuroxime, ROE-AgNPs, or Cefuroxime-ROE-AgNPs

Table 3 shows the MIC and MBC of the solutions against *S. mutans.* Cefuroxime-ROE-AgNPs (1:3) showed MIC (8.5 µg/mL) and MBC (16 µg/mL) followed by ROE-AgNPs, which showed MIC (25 µg/mL) and MBC (35 µg/mL), while cefuroxime showed MIC (32 µg/mL) and MBC (57 µg/mL). The difference was statistically significant between Cefuroxime-ROE-AgNPs and ROE-AgNPs or cefuroxime alone. The decrease of MIC in Cefuroxime-ROE-AgNPs was three-fold compared to the MIC of ROE-AgNPs.

### 3.5. Assessing the Antimicrobial Activity

Table 4 shows the antimicrobial activity of five groups of biomaterials against *S. mutans*. Group D showed the highest inhibition zone (26 mm) as compared to group C (19 mm), and group B (15 mm) and the difference was statistically significant (*p* = 0.001). BIOD and Cefuroxime/BIOD failed to show any antimicrobial activity against *S. mutans*. The present study showed the maximum inhibitory zone of group D against *S. mutans*, with a range of 26 mm at the first 24 h period to 17 mm at the three weeks period.

### 3.6. Determination of Antibacterial Rate Using Colony-Forming Units (CFU)

**Figure 4:**
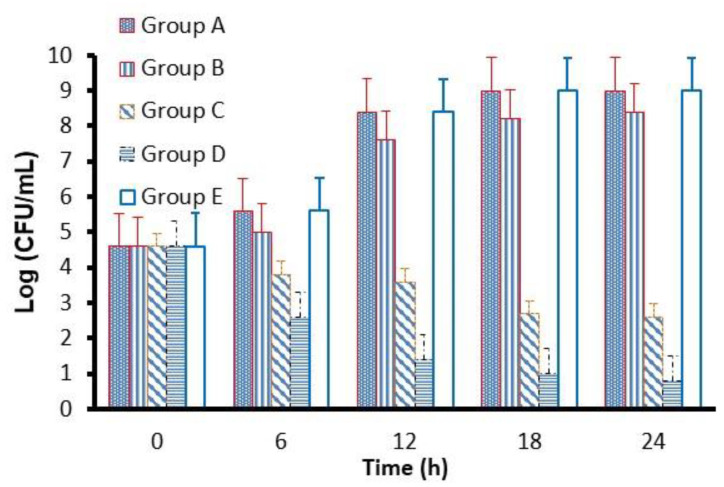
CFU of *S. mutans* cultivated with different samples: (A) Group A BIOD; (B) Group B—0.5% ROE-AgNPs with 99.5% BIOD; Group (C) C—99% BIOD with 1% Cefuroxime-ROE-AgNPs nanontibiotic (1:1); Group (D) D—98% BIOD with 2% Cefuroxime-ROE-AgNPs nanontibiotic (1:3); Group (E) E—98.5% BIOD with 1.5% cefuroxime.

In Figure 4, CFU in the B group is lower than that detected in groups A and E. However, at the same time, the CFU in C and D groups series decreased significantly. The CFU was in the order of group D < group C < group B< group A ≤ group E after 24 h of incubation.

**Figure 5:**
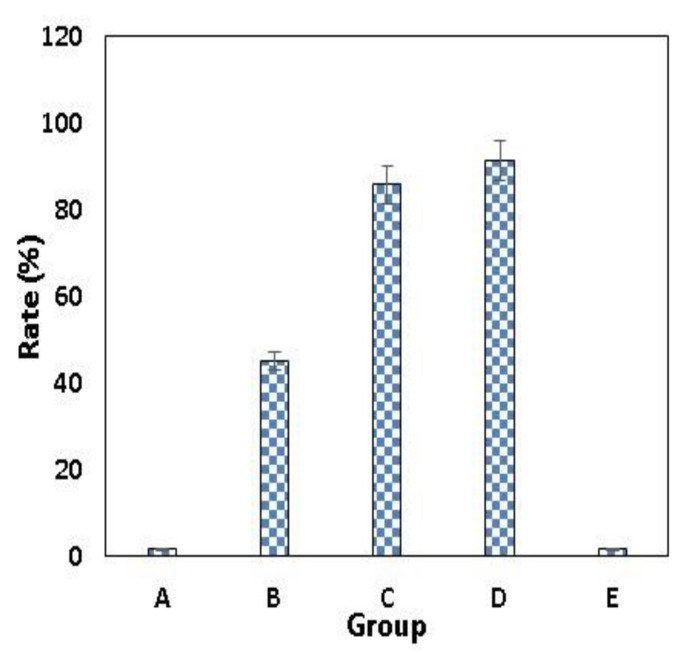
Antibacterial rate (R) of specimens against *S. mutans. R* (%), antibacterial rate. (A) Group A BIOD; (B) Group B—0.5% ROE-AgNPs with 99.5% BIOD; Group (C) C—99% BIOD with 1% Cefuroxime-ROE-AgNPs nanontibiotic (1:1); Group (D) D—98% BIOD with 2% Cefuroxime-ROE-AgNPs nanontibiotic (1:3); Group (E) E—98.5% BIOD with 1.5% cefuroxime.

As shown in Figure 5 and Table 5, the highest antibacterial rates of 85.78% and 91.17% were observed against *S. mutans* in groups C and D, respectively. The order of the antibacterial rate for group D > group C > group B > group A ≥ group E was observed in Table 5 and Figure 5.

### 3.7. Formation of Biofilm and Anti-Biofilm Activity of BIOD Material

**Figure 6:**
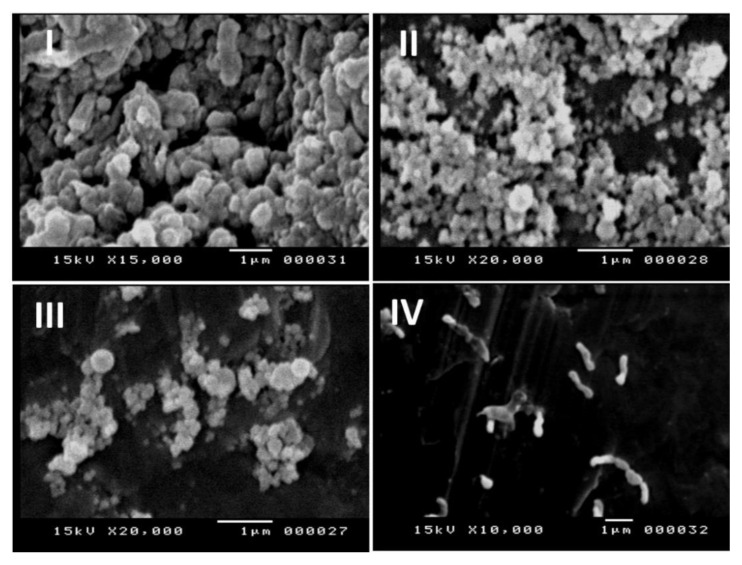
SEM micrographs of the bacteria *S. mutans* after 24 h of incubation time. The micrographs were made on (**I**) Group A—BIOD; (**II**) Group E—98.5% BIOD with 1.5% cefuroxime; (**III**) Group B—99.5% BIOD with 0.5% ROE-AgNPs; (**IV**) D—98% BIOD with 2% Cefuroxime-ROE-AgNPs nanontibiotic (1:3).

The antibiofilm efficiency of BIOD specimens against *S. mutans* is displayed in Figure 6. In a sample of group B (Figure 6III), slight changes in anti-biofilm efficacy and cell morphology were seen as compared to groups A and E (Figure 6I,II) that appeared as an aggregated and clumped cell biomass with normal bacterial cell morphology. In group D (Figure 6IV), the highest anti-biofilm efficiency and changes of cell morphology were seen in addition to the lack of *S. mutans* biofilm adhesion.

## 4. Discussion

Herein, ROE-AgNPs were synthesized using ROE and were conjugated with cefuroxime to form a nanoantibiotic that combined with BIOD to form the novel dental material Cefuroxime-ROE-AgNPs/BIOD. When Cefuroxime-ROE-AgNPs/BIOD antibacterial properties were compared with ROE-AgNPs alone against *S. mutans*, it proved to be stronger. Based on our results, the current study confirmed the success of biosynthesis of ROE-AgNPs from ROE. The color changed to dark brown due to reducing and stabilizing agents such as proteins and phenolic and saponin components of ROE, and this change confirmed the formation of ROE-AgNPs [20]. The change in color was due to surface plasmon resonance (SPR) of ROE-AgNPs. When electric fields of light are directed to ROE-AgNPs, the surface plasmons become excited and begin to resonate. The SPR of ROE-AgNPs authorizes a strong absorption of the incidence light while also allowing some scattering of light; these can be measured using a UV-Vis Spectrometer [40]. In addition, the conjugating efficiency of Cefuroxime-ROE-AgNPs was high, and this indicates that cefuroxime is not lost during preparation and that a lesser amount is needed for therapeutic application [41,42]. The UV-Vis spectroscopy confirmed the formation of ROE-AgNPs and Cefuroxime-ROE-AgNPs at the absorption band of about 400–420 nm due to SPR in ROE-AgNPs [21,40] while it confirmed the presence of a peak at 274 nm for pure cefuroxime. The size of the ROE-AgNPs and Cefuroxime-ROE-AgNPs were in line with the study conducted by Silva et al. [43]. The XRD pattern of ROE-AgNPs and Cefuroxime-ROE-AgNPs showed four major peaks at 2θ values. The strong intensity, narrow width, and sharpness of ROE-AgNPs diffraction peaks in the XRD pattern signalized that the ROE-AgNPs are well crystallized, while broad peaks of Cefuroxime-ROE-AgNPs in the XRD pattern suggested that there was a raise in ROE-AgNPs size owing to the attachment of cefuroxime. The XRD result is in line with that of the recently reported work [42].

FTIR spectra of ROE confirmed the presence of major absorption peaks that revealed a stretching peak of -OH and -NH related to phenolics and proteins in addition to flavonoids and saponins (-OH, -C-H-, and -C-O-C-) [20]. These components were involved in the formation of ROE-AgNPs by reducing Ag^+^ to Ag^0^ [20,43]. Cefuroxime can be interpreted as peaks stretching of (COO^−1^), (-NH) bending vibration, and the band of (-NH) stretching [44].

Upon mixing cefuroxime with ROE-AgNPs, the amide C=O group formed. This was confirmed by the shifting of (-NH) peak of ROE-AgNPs toward longer wave numbers that were previously reported by Hameed et al. [14]. This may be due to the conjugation of a carboxylic group in cefuroxime with the amine group of ROE-AgNPs to form an amide group of Cefuroxime-ROE-AgNPs as well as the presence of a carbonyl group (-C=O) of β lactam stretching [40,41].

### 4.1. In-Vitro Drug Release

Overall, the cefuroxime released from Cefuroxime-ROE-AgNPs/BIOD, group C and D discovered a two-step release: a fast release was displayed at first, approximately 19% and 24%, respectively for 10 days, and this fast release highly depended on the strength of the cefuroxime attachment, and the beginning rapid release enabled a therapeutic dose. In the second step, a prolonged and gradual release profile was observed for the next 96 days. In this prolonged and slow step, the release could be related to Cefuroxime-ROE-AgNPs trapped in the BIOD matrix, and the following long-term release kept this dose during a long period of time that became 56% and 45% of the whole amount, which was obtained from the samples from groups C and D, respectively [45].

### 4.2. Antimicrobial Activity

The MIC and MBC of the Cefuroxime-ROE-AgNPs, ROE-AgNPs, and cefuroxime solutions were detected against *S. mutans.* The difference of MIC and MBC were statistically significant in the case of Cefuroxime-ROE-AgNPs as compared to ROE-AgNPs or cefuroxime alone. The decrease of MIC in Cefuroxime-ROE-AgNPs was three-fold compared to MIC of ROE-AgNPs. In addition, the present study showed a high maximum inhibitory zone of Cefuroxime-ROE-AgNPs against *S. mutans* compared to ROE-AgNPs or cefuroxime alone. The small size of ROE-AgNPs provides them a greater surface area for maximum cefuroxime conjugation as well as high accessibility for resistant bacteria. This is in agreement with the previous studies, which explained that the addition of AgNPs to antibiotics enhances the antibacterial efficacy of clinically approved drugs [22].

Moreover, the CFU in groups C and D of the Cefuroxime-ROE-AgNPs sample series decreased significantly. This may be attributed to the fact that bacterial suspension in the culture medium was directly exposed to Cefuroxime-ROE-AgNPs/BIOD in samples C and D, and that ROE-AgNPs worked as a drug delivery system, which promotes the internalization of the adsorbed cefuroxime, followed by its release. In addition, the various mechanisms of actions of ROE-AgNPs and ionic silver occurred, significantly enhancing *S. mutans* cell death [21,46]. Recently, various drug-conjugated nanoparticles were developed against resistant microbes [21,42].

The order of the antibacterial rate for group D > group C > group B > group A ≥ group E was confirmed. Group C and D ensured 85.78% and 91.17% inhibition of *S. mutant*, respectively. This may be attributed to the fact that the addition of ROE-AgNPs enhanced the antibacterial efficacy of cefuroxime and may explain the superior antimicrobial efficacy for BIOD [21,22] compared to the literature studies [47]. In addition, *R. officinalis* L. was chosen because it effectively reduces bacterial plaque as recorded by Valones et al. [48]. Additionally, *R. officinalis* L. decreases the viability of the biofilm of oral bacteria [49].

Biofilm formation in relation with dental materials was detected in various dental studies such as biofilm formation related to light-curing conditions and finishing and polishing procedures [50,51,52]. Moreover, the biofilm extracellular matrix was difficult to treat by antimicrobial agents. In our study, the antibiofilm efficiency in group D significantly displayed the highest efficacy due to the presence of Cefuroxime-ROE-AgNPs in BIOD cement. Mohamed et al. [53] confirmed the anti-biofilm activity of AgNPs alone and in combination with antibiotics. Moreover, the adherence of bacteria was considered to be an important step in the infection [41]. In group D, bacterial suspension in the culture medium was directly exposed to group D. This led to the prevention of bacterial adhesion and the killing of a bacterium, with the subsequent reduction in CFU count [54]. Additionally, Vazquez-Garcia et al. [47] reported that the addition of AgNPs increased antibacterial efficacy for calcium silicate cements (which is similar to BIOD) and favored the mechanical and physicochemical properties of the dental materials.

Currently, the cefuroxime-ROE-AgNPs/BIOD are successfully formed and exhibit high antimicrobial activity against resistant *S. mutans*. It appears as superior antimicrobial efficacy when compared to other forms (Cefuroxime/BIOD or ROE-AgNPs/BIOD), and this finding adds novelty and significance of the study that can be applied in the dental field to prevent secondary caries. In the future, we will study the antimicrobial activity of this novel material in vivo.

## 5. Conclusions

To conclude the results of the present study, the *R. officinalis* L. extract is a potential natural product for the green biosynthesis of ROE-AgNPs by reduction of silver salts using reducing agents such as phenolics and proteins in addition to flavonoids and saponins present in ROE and for forming ROE-AgNPs. When cefuroxime was conjugated with ROE-AgNPs, it was revived, and it formed Cefuroxime-ROE-AgNPs, which mixed with Biodentine™ to form a novel nanobiomaterial. This material has superior antimicrobial activity against resistant *S. mutant*. Finally, this novel bio-material holds the potential to increase the antimicrobial efficacy against resistant *S. mutans* and will result in the development of cost-effective dental materials for controlling bacterial infections and dental caries in the future.

## Figures and Tables

**Table 1:** Detail of the Biodentine^™^ material.

Powder	Liquid
Tricalcium silicate, dicalcium silicate, calcium carbonate and oxide, iron oxide, and zirconium oxide	Calcium chloride and hydro soluble polymer
(Septodont, Saint-Maur-des-Fosses Cedex, France)	

**Table 2:** Proportion of the experimental materials added to Conventional BIOD cement.

Group Name	Abbreviation	Components
Group A (control)	BIOD	conventional BIOD without any addition.
Group B	ROE-AgNPs/BIOD	(99.5% BIOD with 0.5% ROE-AgNPs, *w*/*w*).
Group C	0.5% Cefuroxime-0.5% ROE-AgNPs/BIOD	(99% BIOD with 1% Cefuroxime-ROE-AgNPs (1:1), *w*/*w*).
Group D	1.5% Cefuroxime-0.5% ROE-AgNPs/BIOD	(98% BIOD with 2% Cefuroxime-ROE-AgNPs (1:3), *w*/*w*).
Group E	Cefuroxime/BIOD	(98.5% BIOD with 1.5% Cefuroxime, *w*/*w*).

**Table 3:** Minimum inhibitory concentration of the solutions against *S. mutant.*

Product	MIC	ANOVA F Value	ANOVA *p* Value	Tukey Post Hoc	MBC (µg/mL)	ANOVA F Value	ANOVA *p* Value	Tukey Post Hoc
Cefuroxime ^a^	0.27 ± 5.3 mg/mL	21.32	0.0001	c < a, b b < a	0.57 ± 7.3 mg/mL	15.42	0.03	c < b, a b < a
AgNPs ^b^	25 ± 2.0 µg/mL				35 ± 1.1 µg/mL			
Cefuroxime-ROE-AgNPs ^c^	8.5 ± 1.3 µg/mL				16 ± 0.8 µg/mL			

^a^—Cefuroxime, ^b^—ROE-AgNPs, ^c^—Cefuroxime-ROE-AgNPs.

**Table 4:** Antimicrobial efficiency of biomaterial against *S. mutans*.

Samples (Groups)	Inhibition Zone (mm)			ANOVA F Value	ANOVA *p* Value	Tukey Post Hoc
	1 Day	2 Weeks	3 Weeks			
a. Group A	8	7	7	NA	NA	NA
b. Group B	15 ± 2.51	12 ± 1.52	10 ± 0.57	8.71	0.03	1 day > 2 and 3rd week
c. Group C	19 ± 1.15	15 ± 0.57	13 ± 1.00	9.36	0.02	1 day > 2 and 3rd week
d. Group D	26 ± 1.52	22 ± 1.52	17 ± 1.15	15.24	0.001	1 day > 2 and 3rd week
e. Group E	8	7	7	NA	NA	NA
ANOVA F Value	19.72	13.27	17.19			
ANOVA *p* Value	0.0001	0.001	0.001			
Tukey post Hoc	d < c, b c < b	d < c, b c < b	d < c, b c < b			

Group A BIOD; (B) Group B—0.5% ROE-AgNPs with 99.5% BIOD; Group (C) C—99% BIOD with 1% Cefuroxime-ROE-AgNPs nanontibiotic (1:1); Group (D) D—98% BIOD with 2% Cefuroxime-ROE-AgNPs nanontibiotic (1:3); Group (E) E—98.5% BIOD with 1.5% cefuroxime.

**Table 5:** Antibacterial rate (*AR*) of specimens against *S. mutans.*

Samples	*AR* (%)
A	1.7 ± 0.10
B	45.07 ± 1.22
C	85.78 ± 0.92
D	91.17 ± 1.12
E	1.8 ± 0.110
F Value ANOVA	19.34
*p* value ANOVA	0.001
Tukey post Hoc	D < B, C C < B

*AR* (%), antibacterial rate. Group A BIOD; (B) Group B—0.5% ROE-AgNPs with 99.5% BIOD; Group (C) C—99% BIOD with 1% Cefuroxime-ROE-AgNPs nanontibiotic (1:1); Group (D) D—98% BIOD with 2% Cefuroxime-ROE-AgNPs nanontibiotic (1:3); Group (E) E—98.5% BIOD with 1.5% cefuroxime.

## Data Availability

Not applicable.

## Sample Availability

Samples of the *Rosmarinus* *officinalis* L. extract are available from the authors.
